# Effect of the age of visual impairment onset on employment outcomes in South Korea: analysis of the national survey on persons with disabilities data

**DOI:** 10.1186/s12889-022-13747-z

**Published:** 2022-08-25

**Authors:** Boyoung Jeon, Heejo Koo, Hye-Jae Lee, Euna Han

**Affiliations:** 1 Department of Health and Medical Information, Myongji College, Seoul, 03656 Republic of Korea; 2 College of Pharmacy, Yonsei Institute of Pharmaceutical Sciences, Yonsei University, Incheon, 21983 Republic of Korea; 3 Department of Environment Health, Korea National Open University, Seoul, 03087 Republic of Korea

**Keywords:** Disabled persons, Visually impaired persons, Age of onset, Employment, South Korea

## Abstract

**Background:**

Opportunities for paid employment provide meaningful ways for those with disabilities to participate in society and achieve financial independence. Although the onset age of disabilities can alter individuals’ attitudes toward accepting their disabilities and their desire for work, the lack of data limits relevant empirical research. The purpose of this study is to examine the effect of the onset age on employment, job security (permanent vs. temporary), and wage level among visually impaired adults in South Korea.

**Methods:**

We used three years of the National Survey on Persons with Disabilities data, 2011, 2014, and 2017, and included 583 participants in this study. We used a logistic regression model for the employment status and a multinomial logistic regression model for job security. We analyzed log monthly wage by a multivariate linear regression model, which subdivided the age groups, with 20–49 years old denoting prime-aged (*n* = 245) and 50–64 years old denoting late-middle-aged (*n* = 338). For each age group, we conducted a sub-analysis by sex.

**Results:**

For prime-aged adults, the employment probability decreased as the age of visual impartment onset increased, and women in particular experienced a lower employment rate for both permanent and temporary jobs when their disability onset age was above 25. However, among permanent employees, monthly wages were higher if the onset age was 25 + compared to when the onset age was 0–5 years old. In late middle-aged adults, adult onset disabilities were associated with higher odds of employment and higher wages for temporary jobs, implying these individuals worked unskilled or manual jobs.

**Conclusions:**

In prime-aged adults, higher monthly wages among permanent employees showed that they were more likely to continue their original work, whereas in late-middle-aged adults, adult-onset disabilities were associated with a higher employment rate and higher wages for temporary jobs, suggesting the need for further investigation into job quality. These findings indicate a need for differentiated policy approaches considering the onset age of visual impairment to improve labor market outcomes throughout individuals’ lifespans.

**Supplementary Information:**

The online version contains supplementary material available at 10.1186/s12889-022-13747-z.

## Background

The opportunity to obtain paid employment is important and meaningful for people with disabilities. In addition to providing a source of income and financial independence, inclusion in the workplace provides companionship, skill development, a sense of community belonging, and positive identity [1, 2]. However, people with disabilities have often been alienated from vocational activities, despite legal support, and visually impaired people are often undervalued in the job market [3].

To encourage the employment of persons with disabilities, the Korean government enforced the Employment Promotion and Vocational Rehabilitation Act in 1990, which imposes a Mandatory Employment Quota of persons with disabilities for the public sector and the private sector of 3.6% and 3.1%, respectively, in 2022, which has been increased gradually from 2% in 1991 [4]. In addition, there are employment support programs for job seekers, such as the Employment Success Package, which provides step-by-step services from pre-employment career counseling to workplace adaptation training in regional offices [4]. Despite these policy efforts, the actual employment rates were 3.00% for the public sector and 2.91% for the private sector in 2020 [5], and the employment rate of people with disabilities was 48% in 2020, whereas that of the general population aged 15–64 years old was 65.8% [6].

In the case of persons with visual impairment, the effect of policies has been limited to those with mild impairment. The employment rate was 42.3% (59.9% of those aged 15–64) for visually impaired people versus 44.4% (64.6% of those aged 15–64) for those with physical disabilities. However, when we focus on the severely disabled group, the employment rate was 18.2% for visually impaired people and 32.1% for physically disabled people among those aged 15 and over [6]. In addition, for wage workers, the ratio of non-regular workers was 64.7% in persons with visual impairments, which was higher than those with physical disabilities (53.2%), implying their lack of job security [6].

Under the unfavorable employment environment for the visually impaired, masseur qualification is designated as a reserved occupation for persons with severe visual impairment through Article 82 of the Medical Act [7], thereby prohibiting those without disability from qualifying as massage therapists. Many persons with severe visual impairment or blindness work in massage-related industries, and vocational rehabilitation services are concentrated in massage-related jobs [8]. Therefore, problems have been raised that the range of occupational choices and individual preferences are not guaranteed when these persons prepare for a new job market [9].

There are 252 thousand persons who have visual impairment in Korea [10]; among them, about 90% have acquired their disability and more than 70% of them have experienced disability onset at the age of 20 or older [11]. The labor market outcomes can differ by the onset age of visual impartment. Those with adult-onset disabilities, who have a disability due to an accident or disease, experience complex difficulties in terms of acceptance of their disabilities, loss of jobs, and social relationships. They have demands for rehabilitation or medical services, economic compensation for treatment expenses, and recovery or renewal of their professional identities [12]*.* If those with adult-onset disabilities are of economically active ages, they strive to accept and adjust to visual impairment by learning new skills and integrating the use of visual aids into their daily routine [13]. They seek to recover their functional restrictions through rehabilitation training, such as digital capabilities [11]. In contrast, those with childhood-onset disabilities have relatively more time to build up skills to prepare for jobs [14], and their disability acceptance is higher than those with adult-onset disabilities [15].

Empirical research on the relationship between age of disability onset and job market outcomes among adults with visual impairment or blindness is limited and has mixed findings [16–19]. The employment rate was higher among visually impaired people with an onset age of under 6 compared with those with an onset age above 6, and wages were lower among people who became visually impaired at age 16 or older [16]. At the same time, individuals with a childhood-onset disability had lower vocational well-being because they often experience educational disruptions due to their disability and the inconvenient nature of many organizational environments [20].

Given the limited and mixed evidence from previous research, this study investigates whether the age of visual impairment onset affects labor market outcomes in South Korea. We explored the relationship between the onset age of visual impairment and various labor market outcomes, including employment, job security, and monthly wages among different age groups: prime-aged adults aged 20–49 and late-middle-aged adults aged 50–64.

## Methods

### Data sources and study participants

We conducted a cross-sectional study using nationwide survey data. The empirical analyses used the National Survey on Persons with Disabilities (NSPD) data for the years 2011, 2014, and 2017. The NSPD contains cross-sectional individual data on disability characteristics, health, socioeconomic status, and discrimination experiences of individuals with disabilities. This survey has been performed by the Korea Ministry of Health and Welfare and the Korea Institute for Health and Social Affairs since 1980 to estimate the prevalence by disability type and to develop national policies for persons with disabilities [21]. The sampling frame of NSPD is based on the nationwide registration census. Among the representative sample of households, the survey interviewed household members who had disabilities. The same sampling methods were used in 2011, 2014, and 2017 [21].

**Fig. 1:**
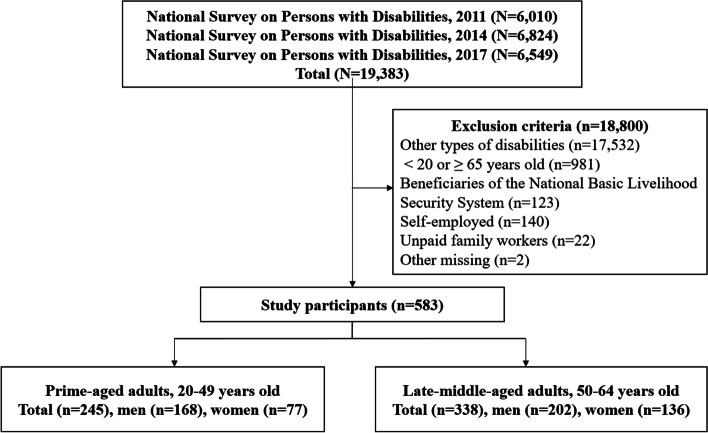
Flowchart of study participants

The pooled NSPD dataset consisted of 19,383 observations, from which this study included adults with visual impairments who were 20–64 years old to focus on the population with potential for economic activities. If a person had co-occurring disabilities, the most severe disability type was defined as their main disability type; therefore, each person had one main disability. Based on the main disability, we excluded people with limb, brain, auditory, kidney, heart, intellectual, developmental, mental, and other disabilities (*n* = 17,532). We excluded people younger than 20 years of age and older than 65 years of age (*n* = 981), beneficiaries of the National Basic Livelihood Security System (*n* = 123), those who were self-employed (*n* = 140), unpaid family workers (*n* = 22), and those whose observations had missing data (*n* = 2). We excluded beneficiaries of the National Basic Livelihood Security System, the income assistance program for households under the designated minimum income, as this program may have an impact on willingness to work [22]. This study focuses on wage earners since there are large gaps in terms of the business scale and income level among the self-employed [23]. We included 583 persons (245 people aged 20–49 years and 338 people aged 50–64 years) (Fig. 1). We obtained ethical approval of this study from the Yonsei University Review Board (IRB No. 202010-HR-2107–01).

### Definition of variables

The three dependent variables were whether the respondent was employed, employment security (unemployed, permanent employee, or temporary employee), and log monthly wages. Monthly wages were standardized to the year 2015 using the GDP deflator. They were measured in units of Korean won (KRW) and converted to the United States dollar (USD) with a conversion rate of 0.847 USD for 1,000 KRW as of December 31, 2015. Monthly wages were transformed using a natural logarithm.

The main independent variable was the age of visual impairment onset (“How old were you when you were first aware of your visual impairment condition?”). The age of visual impairment onset was used in a continuous form or categorized into four groups (0–5 years old, 6–17 years old, 18–24 years old, and 25 years of age or older). These age categories reflect the Korean education system: preschool age (0–5 years old), elementary to high school age (6–11 years old for elementary school, 12–14 years old for middle school, and 15–17 years old for high school), university or military service age (18–24 years old), and working age (25 years of age or older).

This study controlled for the covariates of disability characteristics, sociodemographic factors, health status, and job training experience. Disability severity was included as a disability characteristic. We used the severity index of the Korean Disability Registration system, which reflects visual function and welfare benefits, such as activity assistant. We categorized disability severity as mild (disability severity index 6, i.e., poor visual acuity ≤ 0.02); moderate (severity index 4 or 5, i.e., good visual acuity ≤ 0.1–0.2 or ≤ 10° visual field of both eyes in all directions); and severe (severity index ≤ 3, i.e., good visual acuity ≤ 0.02–0.06 or ≤ 5° visual field of both eyes in all directions) [24]. The variables denoting the cause of visual impairment were congenital or unknown, acquired due to disease, or acquired due to accident.

Sociodemographic factors, including sex, age (continuous), and education (≤ middle school, high school, ≥ college), were controlled for in all estimations. Health conditions were adjusted, including chronic diseases status (presence or absence) and self-rated health status (good versus bad). Job training experience was also controlled for to capture the activities that develop work skills [16] as a dummy indicator to represent vocational training following a disability (in the 2014 and 2017 surveys) or whether a respondent participated in a vocational educational program after the age of 18 (in the 2011 survey). We included dummy year variables to adjust for the year’s fixed effect, such as changes in policies or economic conditions.

### Statistical analysis

We analyzed two age groups, prime-aged adults aged 20–49 [25] and late-middle-aged adults aged 50–64 [26], because the probability of maintaining a job after a health shock is different for those under 50 and those aged 50–64 years old [27, 28]. It has been reported that the average retirement age of wage workers after disability onset is 49.3 years old from their main job, as many people experience involuntary early retirement [29]. After retirement, they continue to engage in economic activities to meet their living expenses [29]. Considering the social context, this study separately analyzed the two groups (20–49 years old and 50–64 years old), and the results of total population (20–64 years old) are presented in the supplementary material (Table S1 and S2). For each age group, we conducted a sub-analysis by sex, as men and women with visual impairments have different job market outcomes [16].

We presented the frequencies and means of general characteristics, employment status, and monthly wages by age and sex. A logistic regression model was used for the binary dependent variable of employment, and multinomial logistic regression was used for the categorical dependent variable of employment security status (unemployed, permanent employee, or temporary employee). For the regression models, we applied a continuous form of the onset age of visual impairment as a main independent variable in Model 1.1 and a categorized form (0–5 years old, 6–17 years old, 18–24 years old, and 25 years of age or older) as a main independent variable in Model 1.2., and we included the same covariates for both. We used multivariate linear regression for the continuous dependent variable of log monthly wages, and we used interaction terms between the onset age of visual impairment and sex to test the differential impact by sex. In summary, Model 2.1 and Model 2.2 included the same covariates, but Model 2.2 added interaction terms between the onset age of visual impairment and sex. We applied heteroscedasticity-robust standard errors in all the regression models.

## Results

### Descriptive statistics

Table 1 shows the general characteristics of the study population. In the prime-aged adults, the average onset age of visual impairment was 17.1 years old. More than half had childhood-onset disabilities (25.7% were at 5 years old or below and 27.3% were at 6–17 years old), while less than one-third had disability onset after the age of 25. The proportion of childhood-onset disabilities was higher in women than men. In late-middle-aged adults, the average onset age of visual impairment was 32.4 years old. More than 66% had disability onset after 25 years old, while 27% had childhood-onset disabilities (10.4% were younger than 6 years old at onset and 16.6% were 6–17 years old).

**Table 1:** General characteristics of the study participants (*n* = 583)

		**Prime-aged adults, 20–49**						**Late-middle-aged adults, 50–64**					
		**Total (*n* = 245)**		**Men (*n* = 168)**		**Women (*n *= 77)**		**Total (*n* = 338)**		**Men (*n* = 202)**		**Women (*n* = 136)**	
		**N**	**%**	**N**	**%**	**N**	**%**	**N**	**%**	**N**	**%**	**N**	**%**
***Key independent variables***													
Onset age of visual impairment													
Continuous	Mean ± SD	17.1 ± 13.1		17.3 ± 12.8		16.6 ± 13.7		32.4 ± 18.2		32.9 ± 18.1		31.5 ± 18.5	
Category	Age 0–5	63	25.7	41	24.4	22	28.6	35	10.4	17	8.4	18	13.2
	Age 6–17	67	27.3	44	26.2	23	29.9	56	16.6	35	17.3	21	15.4
	Age 18–24	38	15.5	32	19.0	6	7.8	23	6.8	15	7.4	8	5.9
	Age 25 +	77	31.4	51	30.4	26	33.8	224	66.3	135	66.8	89	65.4
***Covariates***													
Severity of disability	Mild (Index 6)	175	71.4	125	74.4	50	64.9	235	69.5	143	70.8	92	67.6
	Moderate (Index 4,5)	23	9.4	13	7.7	10	13.0	45	13.3	29	14.4	16	11.8
	Severe (Index 1–3)	47	19.2	30	17.9	17	22.1	58	17.2	30	14.9	28	20.6
Reason for disability	Congenital, unknown	41	16.7	26	15.5	15	19.5	27	8.0	16	7.9	11	8.1
	Acquired due to disease	93	38.0	56	33.3	37	48.1	159	47.0	81	40.1	78	57.4
	Acquired due to accident	111	45.3	86	51.2	25	32.5	152	45.0	105	52.0	47	34.6
Age	Mean ± SD	38.9 ± 7.6		38.2 ± 7.8		40.5 ± 7.0		57.4 ± 4.2		57.0 ± 4.1		58.0 ± 4.4	
Married	Yes	143	58.4	89	53.0	54	70.1	258	76.3	168	83.2	90	66.2
Education	≤ Middle school	31	12.7	19	11.3	12	15.6	173	51.2	87	43.1	86	63.2
	High school	111	45.3	75	44.6	36	46.8	117	34.6	80	39.6	37	27.2
	≥ College	103	42.0	74	44.0	29	37.7	48	14.2	35	17.3	13	9.6
Chronic diseases	Yes	88	35.9	60	35.7	28	36.4	228	67.5	129	63.9	99	72.8
Self-rated health	Bad	45	18.4	27	16.1	18	23.4	139	41.1	72	35.6	67	49.3
Job training	Yes	29	11.8	13	7.7	16	20.8	16	4.7	6	3.0	10	7.4
Survey year	2011	86	35.1	64	38.1	22	28.6	126	37.3	76	37.6	50	36.8
	2014	92	37.6	63	37.5	29	37.7	120	35.5	74	36.6	46	33.8
	2017	67	27.3	41	24.4	26	33.8	92	27.2	52	25.7	40	29.4
***Dependent variables***													
Employment	Yes	166	67.8	128	76.2	38	49.4	169	50.0	118	58.4	51	37.5
Job security^a^	Permanent employee	106	63.9	84	65.6	22	57.9	68	40.2	55	46.6	13	25.5
	Temporary employee	60	36.1	44	34.4	16	42.1	101	59.8	63	53.4	38	74.5
	No	79	32.2	40	23.8	39	50.6	169	50.0	84	41.6	85	62.5
Monthly wage^b^ (Mean ± SD)	All employees	1,774 ± 1,130^c^		1,967 ± 1,171^c^		1,131 ± 666		1,438 ± 1,083		1,653 ± 1,178		941 ± 579	
	Permanent employees	2,124 ± 1,138^c^		2,323 ± 1,155^c^		1,373 ± 668		2,089 ± 1,324		2,238 ± 1,389		1,460 ± 756	
	Temporary employees	1,162 ± 819		1,295 ± 872		796 ± 511		1,000 ± 562		1,143 ± 608		764 ± 374	
**Total**		245	100.0	168	100.0	77	100.0	338	100.0	202	100.0	136	100.0

*Note.*^a^Among the employees. ^b^Among the employees. The monthly wages were adjusted to 2015 using the GDP deflator. Unit: USD (1,000 KRW = about 0.847 USD in December 31, 2015). ^c^There was one non-respondent in the prime-aged adults. Total (*n* = 165), men (*n* = 127) in all employees and men (*n* = 83) in permanent employees

Approximately 19.2% of prime-aged adult respondents had severe disability, and 45.3% acquired their disability due to accidents (51.2% of men and 32.5% of women). More than 87% of the prime-aged adults graduated high school or college, and 11.8% had job training. Among the late-middle-aged adults, 17.2% had severe disability, and the most frequent cause of disability was disease (47.0%), which was more common in women (57.4%) than men (40.1%). More than 51% of the late-middle-aged adults had an educational level of middle school or less, 67.5% had chronic diseases, and 41.1% had poor self-rated health.

The employment rate was 67.8% (76.2% for men and 49.4% for women) for the prime-aged adults and 50% (58.4% for men and 37.5% for women) for the late-middle-aged adults. In terms of job security, 63.9% of prime-aged adults were permanent employees (65.6% of men and 57.9% of women), while 40.2% of the late-middle-aged adult employees were permanent, which was lower for women (25.5%) than men (46.6%). The average monthly wage was 1,774 USD in the prime-aged adults (2,124 USD for permanent employees and 1,162 USD for temporary employees). In the late-middle-aged adults, the average monthly wage was 1,438 USD (2,089 USD for permanent employees and 1,000 USD for temporary employees.) The average wages were higher for men than women for both age groups (Table 1).

### Estimates from regression models

The later the age of visual impartment onset, the lower the odds of employment in prime-aged adults. The impairment onset age was negatively associated with employment in both permanent jobs (adjusted odds ratio [aOR]: 0.23, 95% confidence interval [CI]: 0.08–0.67) and temporary jobs (aOR: 0.35; 95% CI: 0.11–1.10) when the disability onset was during adulthood (25 years of age or older) compared to when it was during childhood (less than 6 years of age). The odds of employment were also lower for those with severe disability, women, and individuals with poor self-rated health, whereas it was higher for respondents who were older or married. Education above the college level significantly increased the possibility of being a permanent employee.

In the late-middle-aged adults, the odds of employment were higher when the disability onset was 25 years or older, especially for temporary employees (aOR: 2.63; 95% CI: 0.97 to 7.09). The odds of employment were also lower for those with severe disability, women, older individuals, and those with poor self-rated health, whereas it was higher for respondents who experienced job training. Job training was significantly associated with a higher likelihood of being a temporary employee (Table 2).

**Table 2:** Impact of the onset age of visual impairment on employment and job security (reference = unemployment)

		**Prime-aged adults, 20–49 (n = 245)**						**Late-middle-aged adults, 50–64 (n = 338)**					
		**Logistic regression**		**Multinomial logistic regression**				**Logistic regression**		**Multinomial logistic regression**			
		**Employment**		**Permanent employees**		**Temporary employees**		**Employment**		**Permanent employees**		**Temporary employees**	
		a**OR** **(95% CI)**	*p-value*	a**OR** **(95% CI)**	*p-value*	a**OR** **(95% CI)**	*p-value*	a**OR** **(95% CI)**	*p-value*	a**OR** **(95% CI)**	*p-value*	a**OR** **(95% CI)**	*p-value*
Onset age of visual impairment													
(ref. = Age 0–5)	Age 6–17	0.57(0.22, 1.46)	0.238	0.53(0.18,1.60)	0.259	0.56(0.18,1.74)	0.318	1.24(0.43,3.54)	0.694	1.19(0.27,5.22)	0.815	1.14(0.34,3.88)	0.829
	Age 18–24	0.75(0.24,2.30)	0.610	0.76(0.21,2.70)	0.668	0.67(0.18,2.54)	0.558	1.35(0.41,4.42)	0.616	1.56(0.30,8.10)	0.594	1.18(0.28,4.93)	0.824
	Age 25 +	0.27(0.10,0.69)	0.007	0.23(0.08,0.67)	0.007	0.35(0.11,1.10)	0.072	2.26(0.93,5.51)	0.072	1.60(0.41,6.15)	0.497	2.63(0.97,7.09)	0.057
Severity of disability	Moderate	0.49(0.14,1.64)	0.246	0.33(0.09,1.31)	0.115	0.77(0.19,3.05)	0.710	0.95(0.45,2.03)	0.903	1.41(0.55,3.64)	0.472	0.85(0.37,1.99)	0.715
(ref. = Mild)	Severe	0.31(0.13,0.74)	0.008	0.20(0.07,0.58)	0.003	0.51(0.19,1.33)	0.167	0.20(0.08,0.50)	0.000	0.17(0.04,0.80)	0.025	0.23(0.09,0.56)	0.001
Reason for disability	Acquired due to disease	1.55(0.59,4.08)	0.375	1.64(0.55,4.87)	0.374	1.40(0.43,4.61)	0.579	1.47(0.51,4.26)	0.475	1.80(0.29,11.15)	0.527	1.33(0.46,3.85)	0.593
(ref. = Congenital, unknown)	Acquired due to accident	1.47(0.57,3.81)	0.424	1.63(0.57,4.71)	0.364	1.34(0.41,4.35)	0.622	1.44(0.54,3.84)	0.470	1.88(0.38,9.41)	0.443	1.24(0.44,3.50)	0.679
Sex	Women	0.17(0.08,0.36)	0.000	0.16(0.07,0.35)	0.000	0.21(0.09,0.51)	0.001	0.38(0.21,0.68)	0.001	0.24(0.11,0.53)	0.000	0.46(0.25,0.84)	0.012
Age	Continuous	1.10(1.05,1.16)	0.000	1.10(1.03,1.16)	0.002	1.11(1.04,1.17)	0.001	0.84(0.78,0.90)	0.000	0.78(0.71,0.85)	0.000	0.87(0.80,0.94)	0.000
Married	Yes	2.17(1.01,4.67)	0.046	3.46(1.48,8.10)	0.004	1.17(0.47,2.90)	0.736	0.56(0.29,1.09)	0.086	0.70(0.28,1.79)	0.458	0.52(0.26,1.04)	0.064
Education	High school	1.46(0.59,3.61)	0.413	1.95(0.66,5.73)	0.224	1.12(0.41,3.05)	0.820	0.60(0.33,1.12)	0.108	0.76(0.34,1.68)	0.495	0.59(0.31,1.15)	0.119
(ref. = ≤ Middle school)	≥ College	1.91(0.71,5.12)	0.198	3.62(1.20,10.88)	0.022	0.81(0.25,2.59)	0.720	0.74(0.38,1.47)	0.392	2.31(0.87,6.12)	0.092	0.25(0.09,0.69)	0.007
Chronic diseases	Yes	0.56(0.27,1.16)	0.120	0.57(0.25,1.29)	0.178	0.54(0.24,1.25)	0.152	1.44(0.78,2.64)	0.244	1.34(0.63,2.87)	0.452	1.51(0.76,3.01)	0.242
Self-rated health	Bad	0.46(0.19,1.10)	0.082	0.52(0.20,1.36)	0.183	0.41(0.13,1.26)	0.119	0.36(0.20,0.65)	0.001	0.14(0.06,0.35)	0.000	0.51(0.27,0.94)	0.032
Job training	Yes	2.22(0.60,8.21)	0.231	1.61(0.35,7.35)	0.541	2.78(0.72,10.76)	0.139	2.85(0.88,9.22)	0.081	0.75(0.06,8.98)	0.820	4.19(1.20,14.67)	0.025
Survey year	2014	0.55(0.25,1.23)	0.144	0.50(0.20,1.22)	0.126	0.61(0.24,1.55)	0.298	1.33(0.70,2.52)	0.386	1.86(0.76,4.55)	0.177	1.19(0.60,2.35)	0.615
(ref. = 2011)	2017	1.10(0.48,2.55)	0.817	1.00(0.39,2.59)	0.998	1.27(0.47,3.47)	0.635	1.51(0.72,3.16)	0.278	2.81(1.09,7.27)	0.033	1.10(0.50,2.43)	0.809
Pseudo R2		0.2222		0.1901				0.1929		0.2154			

*Note.* Robust standard errors were used in the logistic and multinomial regression analyses. aOR means adjusted odds ratio

**Fig. 2:**
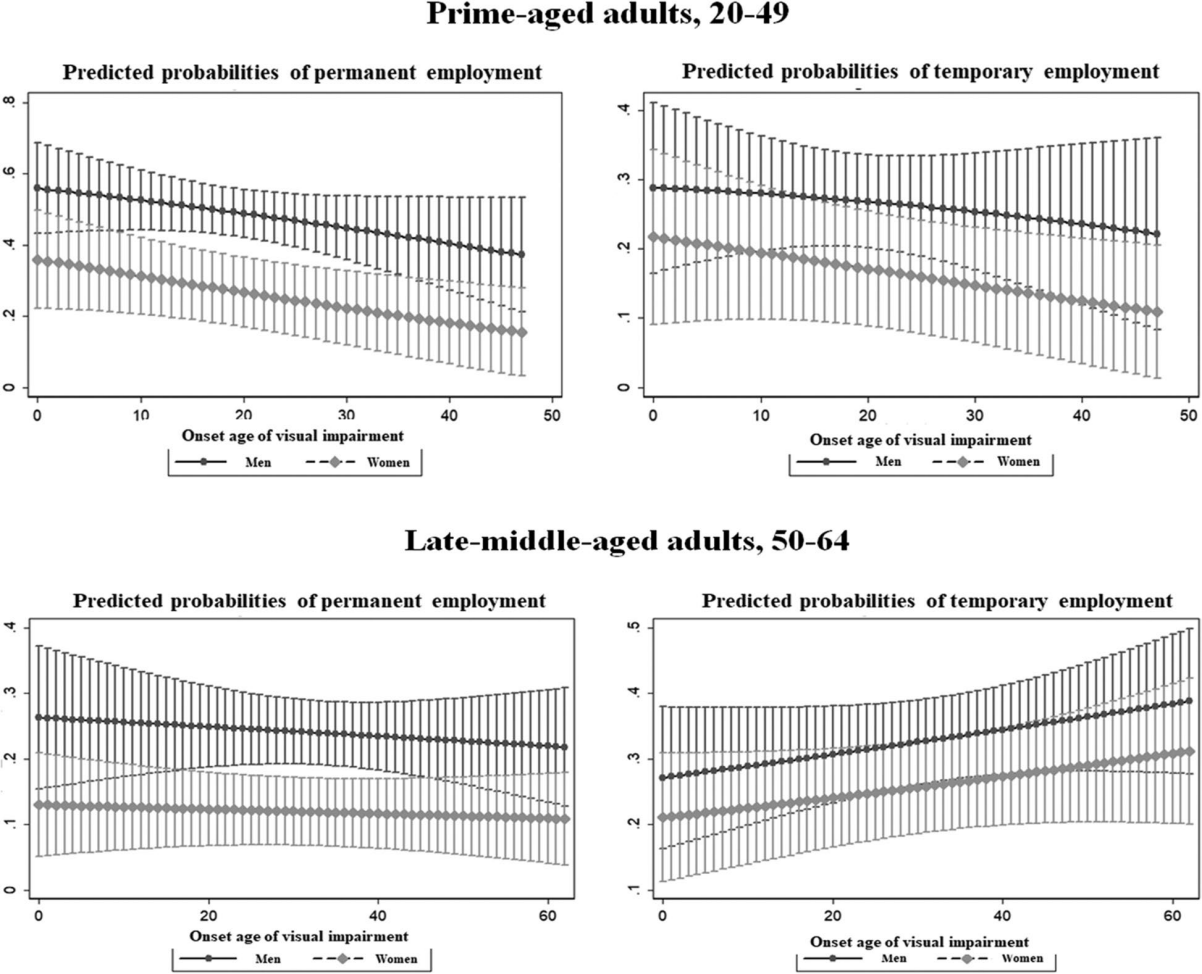
Predicted probability of permanent and temporary employment by the onset age of visual impairment

When we applied subgroup analysis by sex, prime-aged adults demonstrated a lower OR of employment when their age of visual impartment onset was 25 years or older; for men, it was by 0.18 (95% CI: 0.03–0.93), and for women, it was by 0.14 (95% CI: 0.02–0.90). The impact of onset age was significant only for permanent male employees (aOR: 0.14; 95% CI: 0.02–0.77), whereas it was significant for both permanent (aOR: 0.15; 95% CI: 0.17–1.39) and temporary women employees (aOR: 0.05; 95% CI: 0.07–0.75). There was a negative association between disability onset age in continuous form with the likelihood of being a permanent employee (aOR: 0.96; 95% CI: 0.92–1.00) only for women late-middle-aged adults (Table 3). We showed the predicted probabilities of being permanent and temporary employees as the onset age of visual impairment increased by one year using multinomial regression (Fig. 2). It demonstrated an opposing tendency by age group. The average marginal effect of being a temporary employee decreased by -0.0013 for men and -0.0024 for women when the onset age was older in prime-aged adults, whereas it increased by 0.0019 for men and 0.0016 for women when the onset age was older in late-middle-aged adults.

**Table 3:** Impact of the onset age of visual impairment on employment and job security: Subgroup analysis by sex (reference = unemployment)

		**Men**^**a**^						**Women**^**a**^					
		**Logistic regression**^**b**^		**Multinomial logistic regression**^**c**^				**Logistic regression**^**b**^		**Multinomial logistic regression**^**c**^			
		**Employment**		**Permanent employees**		**Temporary employees**		**Employment**		**Permanent employees**		**Temporary employees**	
		**aOR(95%CI)**	*p-value*	**aOR(95%CI)**	*p-value*	**aOR(95%CI)**	*p-value*	**aOR(95%CI)**	*p-value*	**aOR(95%CI)**	*p-value*	**aOR(95%CI)**	*p-value*
**Prime-aged adults, 20–49**													
Onset age of visual impairment													
Model 1.1	Continuous	0.94(0.89, 1.00)	0.044	0.94(0.89,1.00)	0.047	0.96(0.90,1.01)	0.133	0.95(0.90,1.00)	0.073	0.96(0.90,1.02)	0.192	0.92(0.84,1.00)	0.046
Model 1.2 (ref. = Age 0–5)	Age 6–17	0.42(0.10,1.85)	0.252	0.35(0.07,1.79)	0.205	0.49(0.09,2.58)	0.396	0.46(0.09,2.39)	0.359	0.38(0.45,3.73)	0.410	0.53(0.44,2.74)	0.445
	Age 18–24	0.69(0.12,3.88)	0.675	0.65(0.11,4.06)	0.649	0.67(0.09,4.88)	0.696	0.83(0.13,5.35)	0.847	0.46(0.61,6.01)	0.557	0.95(1.14,10.09)	0.963
	Age 25 +	0.18(0.03,0.93)	0.041	0.14(0.02,0.77)	0.025	0.30(0.04,2.04)	0.219	0.14(0.02,0.90)	0.038	0.15(0.17,1.39)	0.095	0.05(0.07,0.75)	0.030
Pseudo R2		Model 1: 0.3720 Model 2: 0.3755		Model 1: 0.2766 Model 2: 0.2836				Model 1: 0.1626 Model 2: 0.1802			Model 1: 0.1603 Model 2: 0.1754		
**Late-middle-aged adults, 50–64**													
Onset age of visual impairment													
Model 1.1	Continuous	1.02(0.99,1.04)	0.144	1.01(0.98,1.04)	0.418	1.02(1.00,1.04)	0.133	0.99(0.97,1.02)	0.556	0.96(0.92,1.00)	0.083	1.00(0.97,1.02)	0.819
Model 1.2 (ref. = Age 0–5)	Age 6–17	1.23(0.24,6.37)	0.804	0.65(0.08,5.25)	0.688	1.60(0.21,12.18)	0.649	1.51(0.22,10.33)	0.675	4.14(0.32,53.37)	0.276	0.93(0.12,7.34)	0.942
	Age 18–24	1.04(0.19, 5.84)	0.965	0.69(0.07,6.31)	0.739	1.28(0.13,12.52)	0.830	0.96(0.14,6.63)	0.964	2.02(0.06,65.04)	0.692	0.80(0.08,8.51)	0.854
	Age 25 +	2.89(0.64,13.05)	0.167	1.16(0.18,7.36)	0.874	4.25(0.66,27.45)	0.128	1.76(0.48,6.41)	0.393	2.15(0.18,25.05)	0.541	1.43(0.39,5.31)	0.591
Pseudo R2		Model 1: 0.1947 Model 2: 0.2078			Model 1: 0.2118 Model 2: 0.2236			Model 1: 0.2270 Model 2: 0.2307			Model 1: 0.2775 Model 2: 0.2741		

*Note.* Robust standard errors were used in the logistic and multinomial regression analyses. aOR means adjusted odds ratio. Model 1.1 and Model 1.2 applied the same covariates in the regression

^a^Number of observations: Prime-aged adults: Men (*n* = 168), women (*n* = 77); Late-middle-aged adults: Men (*n* = 202), women (*n* = 136)

^b^In the logistic regression, disability severity, reason for disability, sex, age, marriage status, education, chronic diseases, self-rated health, job training, and dummy year variables were adjusted

^c^In the multinomial regression, the same covariates were used as in the logistic regression model except for job training due to the small sample size

Log monthly wages were higher by 0.353 (*p* < 0.05) when the onset of disability was 25 or older compared to when the onset of disability was under 6 years old, among prime-aged permanent employees. Women's monthly wages were lower than men's, but there was no significant effect on interaction terms between the onset age of disability and sex. In late-middle-aged permanent employees, the log monthly wages were lower by -0.692 (*p* < 0.1) when the disability onset age was 6–17 years and were lower by -0.641 (*p* < 0.1) when the onset age was 25 years old and over compared to when the disability onset age was under 6 years old. However, the log monthly wages were higher when the onset age of disability was 6 years or older (6–17 years old by 0.419, 18–24 years old by 0.358, and 25 years old and over by 0.448) among late-middle-aged temporary employees. Women's wages were lower than men's, and the main effect of the onset age of disability was mitigated through the interaction term between the onset age of disability and sex (Table 4).

**Table 4:** Impact of onset age of visual impairment on log monthly wages^a,b^

		**Prime-aged adults, 20–49**						**Late-middle-aged adults, 50–64**					
		**All employees**		**Permanent employees**		**Temporary employees**		**All employees**		**Permanent employees**		**Temporary employees**	
		**Model 2.1**	**Model 2.2**	**Model 2.1**	**Model 2.2**	**Model 2.1**	**Model 2.2**	**Model 2.1**	**Model 2.2**	**Model 2.1**	**Model 2.2**	**Model 2.1**	**Model 2.2**
		**Coef. (SE)**	**Coef. (SE)**	**Coef. (SE)**	**Coef. (SE)**	**Coef. (SE)**	**Coef. (SE)**	**Coef. (SE)**	**Coef. (SE)**	**Coef. (SE)**	**Coef. (SE)**	**Coef. (SE)**	**Coef. (SE)**
Onset age of visual impairment													
(ref. = Age 0–5)	Age 6–17	-0.040 (0.113)	-0.038 (0.139)	0.086 (0.105)	0.084 (0.149)	-0.003 (0.242)	0.138 (0.301)	0.074 (0.269)	-0.335 (0.513)	-0.692* (0.376)	-0.902** (0.309)	0.419* (0.211)	1.062** (0.484)
	Age 18–24	0.014 (0.145)	0.068 (0.149)	0.106 (0.136)	0.224 (0.151)	0.303 (0.376)	0.255 (0.401)	0.382 (0.305)	0.197 (0.555)	-0.127 (0.452)	-0.070 (0.398)	0.358* (0.212)	1.165** (0.459)
	Age 25 +	0.102 (0.136)	0.134 (0.156)	0.353** (0.126)	0.435** (0.158)	0.128 (0.244)	0.266 (0.268)	0.118 (0.259)	-0.227 (0.506)	-0.641* (0.344)	-0.890** (0.274)	0.448** (0.202)	1.122** (0.427)
Sex	Women	-0.530** (0.118)	-0.450* (0.230)	-0.660** (0.108)	-0.424** (0.183)	-0.339* (0.193)	-0.261 (0.362)	-0.381** (0.110)	-0.943* (0.522)	-0.313* (0.176)	-1.255** (0.392)	-0.386** (0.130)	0.355 (0.447)
Interaction terms													
	Age 6–17*Women		-0.026 (0.288)		-0.069 (0.245)		-0.177 (0.465)		0.855 (0.530)		1.209** (0.415)		-0.633 (0.525)
	Age 18–24*Women		-0.303 (0.570)		-0.990** (0.474)		0.628 (0.571)		-0.009 (0.662)		-0.439 (0.592)		-1.386* (0.717)
	Age 25 + *Women		-0.145 (0.305)		-0.372 (0.234)		-0.575 (0.535)		0.597 (0.537)		1.146** (0.417)		-0.847* (0.504)
R2		0.4260	0.4292	0.5841	0.6246	0.4186	0.4535	0.4156	0.4409	0.4265	0.5188	0.3884	0.4119
N		165	165	105	105	60	60	169	169	68	68	101	101

*Note.* Robust standard errors were used in the linear regression analyses. Model 2.1 and Model 2.2 applied the same covariates, but Model 2.2 added interaction terms between the onset age of visual impairment and sex

^a^For “monthly wages,” the monthly wages were adjusted to 2015 values using the GDP deflator

^b^In the regression, we adjusted for disability severity, reason for disability, age, marriage status, education, chronic diseases, self-rated health, job training, and dummy year variables

*p-value < 0.1, **p-value < 0.05

## Discussion

This study investigated the relationship between the onset age of visual impartment and labor market outcomes, including employment, job security, and log monthly wages, among prime-aged adults (those 20–49 years of age) and late-middle-aged adults (those 50–64 years of age). People with adult-onset disabilities showed lower employment rate than those with childhood-onset disabilities, when women had adult-onset disabilities, they were more likely to experience hardness in getting a permanent or temporary job within the prime-aged adults, while persons with adult-onset disabilities were more likely to work for temporary jobs than those with childhood-onset disabilities within the late-middle-aged adults.

The wage-earning employment rates in those with visual impairment were 67.8% among prime-aged adults and 50% among late-middle-aged adults, whereas the corresponding rates in the entire population as of 2015 were 54.9% (73.0% for men and 53.0% for women) among prime-aged adults and 39.1% (70.7% for men and 37.7% for women) among late-middle-aged adults after excluding self-employed and unpaid family workers [30]. The higher employment rate of persons with visual impairment compared to the entire population is likely due to there being a higher portion of men among the visually impaired in the present study than in the general population (68.6% versus 50.4% among adults aged 20–49; 59.8% versus 49.8% among adults aged 50–64) [30], and it is also caused by the exclusion of the beneficiaries of the National Basic Livelihood Security System (n = 123), which is an income subsidy program, as it is reported that 95% of them are not engaged in work [22].

However, job security and average monthly wages were lower for those with visual impairments than for the entire population. Among those with visual impairments, 63.9% of prime-aged adults and 40.2% of late-middle-aged adults were permanent employees. These are lower than the corresponding proportion (74.1% among adults aged 20–49; 65.4% among adults aged 50–59) of the entire population [31]. The average monthly wage was 1,774 USD for prime-aged adults and 1,438 USD for late-middle-aged adults, which were lower than the average for wage workers (2,053 USD) for the entire population above 15 years of age [32].

Our study showed that the employment rate was lower for those with adult-onset disabilities among prime-aged adults, especially when a person experienced onset of visual impairment over the age of 25 years old. If a person experienced visual impairment in the middle of their lifespan, they might find it difficult to continue working, maintain the same level of job performance, or obtain new job opportunities [14]. In contrast, those with childhood-onset disabilities have relatively diverse opportunities to acquire skills required in the job market, such as digital skills and social skills [14]. Persons with adult-onset disabilities might need time to accept their disability [15], and the delayed time for recovering their functional limitations and social relationship would hold back their employment. It is known that people with higher levels of disability acceptance are more likely to want to participate in the labor market and have a higher employment rate or re-employment rate [33–35], therefore, support for psychological recovery can be helpful [36]. In addition, those with adult-onset disabilities face a lack of adequate rehabilitation programs for returning to work or getting a decent job [37], and they do not have many job choices other than massage-related work or simple labor jobs [9]. As there is a growing demand for skill trainings to reflect the rapidly changing occupations and a variety of job types, policy efforts are needed to develop vocational rehabilitation programs, including recent job trends, professional career counseling and job match, group counseling services or individual case management [36]. These efforts may have effect on satisfying the demands of job seekers who want to work in occupations other than massage-related work [36].

We also demonstrated that the disability severity was significantly associated with the likelihood of permanent employment. Both prime-aged adults and late-middle-aged adults with severe visual impairment had a lower likelihood of having a permanent job. It is known that the employment rate of the severely disabled group in persons with visual impairment is much lower [6], and our result proved that the gap came from permanent employment. Some employers prefer hiring people with mild disabilities because those with severe disabilities need assistive devices or personal support [18], and it may create a barrier when those with severe disabilities try to find an adequate workplace [38]. Even if a severely visually impaired person finds a job, some of them are assigned inappropriate tasks due to a company does not carefully consider their characteristics or prepare the assistive devices. In addition, the unmatched tasks with their ability threatens their work-related identity, and the loss of the identity can be linked to quit a job [39]. Under the current Mandatory Employment Quota system, double rate is applied for the severely disabled, when the mandatory employment rate for the disabled is calculated, since 2010 [40]. However, additional policy for support the retention of employment rate is also important, through wages, appropriate work arrangements, installation of convenience facilities, and personal assistance [41].

Education level are known human capital factors of economic independence [42], and their effect was only significant in permanent employment among prime-aged adults. However, the education effect was opposite for temporary employment, especially among the late-middle-aged adults. The likelihood of temporary employment became significantly lower for the college or higher education group, which suggests that their preexisting knowledge or skills would not be adequately utilized and the job quality would not be suitable for this group. Part-time jobs can be a complementary means for people with visual impairment, especially who have received a high level of education but working hours are limited due to health reasons or other priorities in their lifecycle, providing opportunities for economic independence and broad social participation [43, 44].

When the onset age of disability was 25 years or older, the monthly wage level for permanent workers was higher, although the odds of permanent employment were lower. It is possible to continue to work even after occurrence of adult-onset disabilities, if job security is guaranteed [33] and wages can increase over time [45]. The lower income level of people with childhood-onset disabilities may be caused by low job quality [46]. Young persons with visual impairment often face challenges in mobility and interpersonal relationships, and their working environment may not be disability-friendly [47]. Our analyses also showed that those with a disability onset age of over 25 years old showed higher levels of job satisfaction (88.5%) than those with a disability onset age of under 6 years old (85.2%), 6–17 years old (81.3%), or 18–24 years old (71.4%) among permanent employees aged 20–49 (Table S3 in the supplementary material).

The subgroup analysis by sex showed that women experienced a lower employment rate when their onset age of disability was over 25 for both permanent and temporary jobs, whereas men had difficulties only in permanent jobs. Women with disabilities have more socio-economically disadvantages than men [48], and the present study emphasized that adult-onset disabilities can be an additional barrier for women, even after adjusting for marital status or educational level. Women’s jobs are less likely to be stable, making it difficult for them to return to work. Women might also face negative employer attitudes when trying to obtain a job after visual impairment. They may face even more obstacles if they have children under the age of 16 [49]. These imply that job flexibility, e.g. time-selective jobs, and vocational training programs to improve their work capabilities rather than simple tasks are needed for women with adult-onset visual impairment [44, 49].

“Aging with disability” and “disability with aging” are mixed in late-middle-aged adults aged 50–64. Many people in this age group retire early from their original work or transition to other jobs [50]. According to our study, the odds of employment were higher when the onset age of disability was 25 years or older, especially for temporary employment. Temporary jobs that do not require specialized knowledge or skills may be easy to find for those with adult-onset disabilities.

The effect of the onset age of disability on wages was reversed for permanent workers and temporary workers within late-middle-aged adults. The higher the onset age of disability, the lower the wages in permanent workers, whereas the opposite was true in temporary workers. Permanent jobs tend to provide more flexible working conditions, allowing employees with adult-onset disabilities to adjust their working conditions without fear of losing their jobs. Alternatively, those with adult-onset disabilities in permanent jobs are likely in managerial positions, where they have self-determination on their working conditions and are able to reduce their working time, work intensity, and salary. In the case of temporary jobs, those with adult-onset disabilities would participate in non-skillful work or manual labor, spend long hours at work, and earn relatively higher wages. On the other hands, individuals with childhood-onset disabilities may also have experienced pre-labor market differences when the late-middle-aged adults were being educated in the 1960s–70 s [14]. There has been social prejudice against those with disabilities [51], so persons with childhood-onset disabilities would have had difficulties in accumulating labor skills or qualifications while growing up. This, in turn, could lower their employment rate and wages.

Job training was found to increase the likelihood of temporary employment in late-middle-aged adults. Approximately 8% of those aged 15 years or older in the entire population had job training experience [52], while 11.8% of prime-aged adults with visual impairment and less than 5% of late-middle-aged adults with visual impairment had such training. Job training programs are not specialized enough for those with visual impairments, which may restrict these persons to assembly work or massage acupressure training, regardless of education status [8]. Further studies need to assess the role of job training or occupational rehabilitation programs in those with visual impairment, considering the effect of onset age of disability and the severity of the disability.

There are several limitations to this study. First, people with mild disabilities accounted for approximately 70% of the study sample. This study therefore does not appropriately represent people with severe visual impairment, such as blindness. It may also underestimate the effect of onset age of visual impairment on labor market outcomes. In addition, although we used a representative national survey data of individuals with disabilities, the final sample size was not enough to generalize for those with visual impairment. Further studies need to reflect the situations of severe visual impairment using the revised disability severity index of the Korean Disability Registration system [53] and a bigger size survey or cohort study for examining the labor market of individuals with visual impairment would be needed. Second, job quality was not fully considered, as this study only considered employment and income security. Further research should consider job quality, such as development possibilities, work conditions, relationships, and employers’ understanding of disability [54].

## Conclusions

This study demonstrates that people with adult-onset disabilities had lower odds of employment and that women with adult-onset disabilities faced a disadvantage in obtaining permanent and temporary jobs. Among late-middle-aged adults, adult-onset disabilities were associated with a higher employment rate and higher wages for temporary jobs. Many temporary jobs involve unskilled or manual works, suggesting the need for further investigation into job quality. These findings indicate the need for differentiated policy approaches considering the age of disability onset to improve labor market outcomes throughout the lifespans of those with visual impairments.

## Supplementary Information

**Additional file 1: Table S1. **General characteristics of the study participants among the adults aged 20–64 (*n* = 583). **Table S2. **Impact of the onset age of visual impairment on employment, job security, and log monthly wages among the adults aged 20–64.** Table S3. **Job satisfaction according to the onset age of visual impairment.

## Data Availability

The datasets analyzed during the current study are available in the Korean Health and Welfare Data Portal at [https://data.kihasa.re.kr/]. The data can be downloaded after a researcher applies and obtains permission to utilize a dataset from the Korea Institute for Health and Social Affairs.

## Abbreviations

NSPD: The National Survey on Persons with Disabilities
KRW: Korean won
USD: United States dollar
aOR: Adjusted odds ratio
95% CI: 95% Confidence interval
