# Supplementary material for: Effect of the age of visual impairment onset on employment outcomes in South Korea: analysis of the national survey on persons with disabilities data
Source: BMC Public Health. 2022 Aug 25;22:1613. doi: 10.1186/s12889-022-13747-z (PMC9404557; doi:10.1186/s12889-022-13747-z)
Supplement: Supplementary file 1 — Additional file 1: Table S1. General characteristics of the study participants among the adults aged 20–64 (n = 583). Table S2. Impact of the onset age of visual impairment on employment, job security, and log monthly wages among the adults aged 20–64. Table S3. Job satisfaction according to the onset age of visual impairment. [file 12889_2022_13747_MOESM1_ESM.docx]

**Supplementary material**

**Table S1. General characteristics of the study participants among the adults aged 20–64 (n = 583)**

|  |  | **N** | **%** |
| --- | --- | --- | --- |
| ***Key independent variables: Onset age of visual impairment*** | |  |  |
| Continuous | Mean±SD | 26.0±17.9 | |
| Category | Age 0–5 | 98 | 16.8 |
|  | Age 6–17 | 123 | 21.1 |
|  | Age 18–24 | 61 | 10.5 |
|  | Age 25+ | 301 | 51.6 |
| ***Covariates*** |  |  |  |
| Severity of disability | Mild (Index 6) | 410 | 70.3 |
|  | Moderate (Index 4,5) | 68 | 11.7 |
|  | Severe (Index 1–3) | 105 | 18.0 |
| Reason for disability | Congenital, unknown | 68 | 11.7 |
|  | Acquired due to disease | 252 | 43.2 |
|  | Acquired due to accident | 263 | 45.1 |
| Sex | Male | 370 | 63.5 |
|  | Female | 213 | 36.5 |
| Age | Mean±SD | 49.7±10.9 | |
| Married | Yes | 401 | 68.8 |
| Education | ≤Middle school | 204 | 35.0 |
|  | High school | 228 | 39.1 |
|  | ≥College | 151 | 25.9 |
| Chronic diseases | Yes | 316 | 54.2 |
| Self-rated health | Bad | 184 | 31.6 |
| Job training | Yes | 45 | 7.7 |
| Survey year | 2011 | 212 | 36.4 |
|  | 2014 | 212 | 36.4 |
|  | 2017 | 159 | 27.3 |
| ***Dependent variables*** | |  |  |
| Employment | Yes | 335 | 57.5 |
| Job security^a^ | Permanent employee | 174 | 51.9 |
|  | Temporary employee | 161 | 48.1 |
|  | No | 248 | 42.5 |
| Monthly wage^b^ | All employees (n=334) | 1,604 ±1,118 | |
| (Mean±SD) | Permanent employees^c^ (n=173) | 2,110 ±1,211 | |
|  | Temporary employees (n=161) | 1,060 ±671 | |
| Total |  | 583 | 100.0 |
| *Note.* ^a^Among the employees. ^b^Among the employees. The monthly wages were adjusted to 2015 using the GDP deflator. Unit: USD (1,000 KRW = about 0.847 USD in December 31, 2015). ^c^There was one non-respondent. | | | |

**Table S2. Impact of the onset age of visual impairment on employment, job security, and log monthly wages among the adults aged 20–64**

|  |  | Logistic regression | | Multinomial logistic regression | | | | Multivariate linear regression on log monthly wages | | | | | |
| --- | --- | --- | --- | --- | --- | --- | --- | --- | --- | --- | --- | --- | --- |
|  |  | Employment | | Permanent employees | | Temporary employees | | All employees | | Permanent employees | | Temporary employees | |
|  |  | aOR(95% CI) | *p-value* | aOR(95% CI) | *p-value* | aOR  (95% CI) | *p-value* | Coef. (SE) | *p-value* | Coef. (SE) | *p-value* | Coef. (SE) | *p-value* |
| Onset age of visual impairment | | | | | | | | | | | | | |
| (ref.= Age 0–5) | Age 6–17 | 1.04(0.56,1.92) | 0.909 | 1.13(0.54,2.36) | 0.745 | 0.96(0.45,2.06) | 0.925 | -0.042(0.108) | 0.699 | -0.094(0.122) | 0.444 | 0.121(0.162) | 0.458 |
|  | Age 18–24 | 1.39(0.6,2.98) | 0.395 | 1.65(0.67,4.04) | 0.272 | 1.18(0.47,2.97) | 0.726 | 0.098(0.130) | 0.454 | 0.002(0.147) | 0.987 | 0.264(0.212) | 0.215 |
|  | Age 25+ | 1.28(0.72,2.29) | 0.403 | 1.08(0.54,2.17) | 0.830 | 1.53(0.77,3.04) | 0.224 | 0.042(0.116) | 0.719 | -0.016(0.133) | 0.903 | 0.271(0.166) | 0.105 |
| Severity of disability | Moderate | 0.80(0.44,1.44) | 0.454 | 0.82(0.41,1.64) | 0.575 | 0.82(0.42,1.62) | 0.575 | -0.118(0.102) | 0.249 | -0.077(0.124) | 0.535 | -0.143(0.163) | 0.382 |
| (ref.= Mild) | Severe | 0.32(0.19,0.54) | 0.000 | 0.26(0.13,0.53) | 0.000 | 0.39(0.21,0.70) | 0.002 | -0.285(0.121) | 0.019 | -0.368(0.132) | 0.006 | -0.162(0.152) | 0.286 |
| Reason for disability | Acquired due to disease | 1.10(0.57,2.13) | 0.769 | 1.17(0.52,2.62) | 0.701 | 1.00(0.45,2.21) | 0.999 | -0.009(0.136) | 0.946 | -0.222(0.119) | 0.065 | 0.168(0.213) | 0.433 |
| (ref.=Congenital, unknown) | Acquired due to accident | 1.28(0.67,2.46) | 0.459 | 1.40(0.65,3.04) | 0.392 | 1.14(0.52,2.49) | 0.751 | 0.020(0.137) | 0.886 | -0.159(0.125) | 0.206 | 0.069(0.222) | 0.756 |
| Sex | Female | 0.40(0.27,0.60) | 0.000 | 0.30(0.18,0.50) | 0.000 | 0.48(0.31,0.75) | 0.001 | -0.392(0.076) | 0.000 | -0.453(0.093) | 0.000 | -0.279(0.099) | 0.006 |
| Age | Continuous | 0.98(0.96,1.00) | 0.076 | 0.97(0.94,0.99) | 0.014 | 0.99(0.96,1.01) | 0.363 | -0.001(0.004) | 0.863 | 0.002(0.006) | 0.694 | -0.004(0.006) | 0.555 |
| Married | Yes | 1.21(0.78,1.88) | 0.393 | 2.17(1.17,4.01) | 0.013 | 0.84(0.52,1.36) | 0.477 | 0.233(0.069) | 0.001 | 0.138(0.080) | 0.087 | 0.172(0.099) | 0.084 |
| Education | High school | 0.89(0.56,1.40) | 0.606 | 1.27(0.69,2.31) | 0.443 | 0.77(0.46,1.27) | 0.304 | 0.300(0.092) | 0.001 | 0.204(0.111) | 0.067 | 0.260(0.125) | 0.039 |
| (ref.=≤Middle school) | ≥College | 1.21(0.71,2.08) | 0.483 | 2.80(1.44,5.44) | 0.002 | 0.47(0.24,0.91) | 0.024 | 0.629(0.103) | 0.000 | 0.485(0.119) | 0.000 | 0.208(0.194) | 0.284 |
| Chronic diseases | Yes | 0.87(0.57,1.32) | 0.513 | 0.80(0.48,1.32) | 0.381 | 0.96(0.59,1.57) | 0.885 | -0.013(0.076) | 0.863 | 0.030(0.087) | 0.730 | -0.074(0.118) | 0.530 |
| Self-rated health | Bad | 0.43(0.28,0.68) | 0.000 | 0.31(0.17,0.56) | 0.000 | 0.52(0.32,0.87) | 0.012 | -0.141(0.090) | 0.117 | -0.120(0.122) | 0.327 | -0.017(0.130) | 0.898 |
| Job training | Yes | 1.95(0.85,4.46) | 0.113 | 1.07(0.35,3.27) | 0.911 | 2.83(1.22,6.58) | 0.015 | -0.008(0.126) | 0.952 | 0.351(0.117) | 0.003 | -0.065(0.142) | 0.647 |
| Survey year | 2014 | 0.88(0.56,1.37) | 0.563 | 0.94(0.54,1.63) | 0.829 | 0.84(0.51,1.40) | 0.502 | 0.177(0.087) | 0.041 | 0.138(0.097) | 0.158 | 0.249(0.136) | 0.070 |
| (ref.= 2011) | 2017 | 1.23(0.75,2.02) | 0.407 | 1.56(0.85,2.84) | 0.148 | 1.05(0.59,1.86) | 0.867 | 0.341(0.094) | 0.000 | 0.239(0.093) | 0.011 | 0.449(0.156) | 0.005 |
| Pseudo R2 or R-squared | | Pseudo R2 = 0.1302 | | Pseudo R2 = 0.1551 | | | | R-squared = 0.3758 | | R-squared = 0.3547 | | R-squared = 0.2944 | |
| N | | 583 | | 583 | | | | 334 | | 173 | | 161 | |
| *Note.* Robust standard errors were used in the logistic and multinomial regression analyses. aOR means adjusted odds ratio. | | | | | | | | | | | | | |

**Table S3. Job satisfaction according to the onset age of visual impairment**

|  |  | **Prime-aged adults, 20–49** | | | | | |  | **Late-middle-aged adults, 50–64** | | | | | |
| --- | --- | --- | --- | --- | --- | --- | --- | --- | --- | --- | --- | --- | --- | --- |
|  |  | **All employees**  **(n=166)** | | **Permanent employees (n=106)** | | **Temporary employees (n=60)** | |  | **All employees**  **(n=169)** | | **Permanent employees (n=68)** | | **Temporary employees (n=101)** | |
|  |  | **Unsatisfied**  **N(row %)** | **Satisfied**  **N(row %)** | **Unsatisfied**  **N(row %)** | **Satisfied**  **N(row %)** | **Unsatisfied**  **N(row %)** | **Satisfied**  **N(row %)** |  | **Unsatisfied**  **N(row %)** | **Satisfied**  **N(row %)** | **Unsatisfied**  **N(row %)** | **Satisfied**  **N(row %)** | **Unsatisfied**  **N(row %)** | **Satisfied**  **N(row %)** |
| Onset age of visual impairment | | | | | | | | | | | | | | |
|  | Age 0–5 | 10(23.3) | 33(76.7) | 4(14.8) | 23(85.2) | 6(37.5) | 10(62.5) |  | 3(25.0) | 9(75.0) | 0(0.0) | 4(100.0) | 3(37.5) | 5(62.5) |
|  | Age 6–17 | 14(29.8) | 33(70.2) | 6(18.8) | 26(81.3) | 8(53.3) | 7(46.7) |  | 6(21.4) | 22(78.6) | 0(0.0) | 15(100.0) | 6(46.2) | 7(53.8) |
|  | Age 18–24 | 10(33.3) | 20(66.7) | 6(28.6) | 15(71.4) | 4(44.4) | 5(55.6) |  | 6(54.5) | 5(45.5) | 3(50.0) | 3(50.0) | 3(60.0) | 2(40.0) |
|  | Age 25+ | 16(34.8) | 30(65.2) | 3(11.5) | 23(88.5) | 13(65.0) | 7(35.0) |  | 45(38.1) | 73(61.9) | 9(20.9) | 34(79.1) | 36(48.0) | 39(52.0) |
| Total | | 50(30.1) | 116(69.9) | 19(17.9) | 87(82.1) | 31(51.7) | 29(48.3) |  | 60(35.5) | 109(64.5) | 12(17.6) | 56(82.4) | 48(47.5) | 53(52.5) |
